# Randomized clinical trial of continuous transversus thoracis muscle plane block for patients undergoing open heart valve replacement surgery

**DOI:** 10.1111/jcmm.18184

**Published:** 2024-03-20

**Authors:** Yanping Zhan, Lei Li, Shibiao Chen, Yongbao Peng, Yang Zhang

**Affiliations:** ^1^ Department of Anaesthesiology First Affiliated Hospital of Nanchang University Nanchang China; ^2^ Department of Anaesthesiology Jiangxi Maternal and Child Health Hospital Nanchang China

**Keywords:** continuous transversus thoracis muscle plane blocks, open cardiac surgery, post‐operative pain, sufentanil, the length of hospital stay

## Abstract

The optimal analgesia regimen after open cardiac surgery is unclear. The aim of this study was to investigate the beneficial effects of continuous transversus thoracis muscle plane (TTMP) blocks initiated before surgery on open cardiac surgery outcomes. A group of 110 patients were randomly allocated to either receive bilateral continuous TTMP blocks (TTP group) or no nerve block (SAL group). The primary endpoint was post‐operative pain at 4, 8, 16, 24, 48 and 72 h after extubation at rest and exercise. The secondary outcome measures included analgesia requirements (sufentanil and flurbiprofen axetil administration), time to extubation, incidence of reintubation, length of stay in the ICU, incidence of post‐operative nausea and vomiting (PONV), time until return of bowel function, time to mobilization, urinary catheter removal and length of hospital stay. The length of stay in the ICU and length of hospital stay were significantly longer in the SAL group than in the TTP group. NRS scores at rest and exercise were significantly lower in the TTP group than in the SAL group at all time points. The TTP group required significantly less intraoperative and post‐operative sufentanil and post‐operative dynastat consumption than the SAL group. Time to extubation, time to first flatus, time until mobilization and time until urinary catheter removal were significantly earlier in the TTP group than in the SAL group. The incidence of PONV was significantly lower in the TTP group. Bilateral continuous TTMP blocks provide effective analgesia and accelerate recovery in patients undergoing open heart valve replacement surgery.

## 1 INTRODUCTION

Open cardiac surgery is still the standard for more than 1.5 million patients worldwide undergoing surgery for heart disease every year.^1^ Patients undergoing cardiac surgery may experience severe acute post‐operative pain,^2^, ^3^ and the mid‐sternum is the main site of pain after cardiac surgery.^4^ Patients who undergo cardiac surgery and experience poor post‐operative analgesia are less satisfied and endure delirium, delirium, hypertension, tachycardia, arrhythmias, hyperglycaemia, bronchial secretion stasis, atelectasis and pneumonia.^5^ High‐dose opioids are most commonly used to alleviate pain in patients undergoing cardiac surgery, but opioids can cause adverse effects, including nausea and vomiting, pruritus, respiratory depression, delayed tracheal extubation, sedation and ileus.^6^

Thoracic epidural anaesthesia and paravertebral blocks can provide effective analgesia for patients undergoing heart surgery and reduce opioid use in cardiac surgical patients,^7^ but adverse effects, such as pneumothorax and injury to the spinal cord, sympathectomy‐induced hypotension and potentially life‐threatening epidural haematoma caused by post‐operative coagulopathy and anticoagulant use have limited the wide application of regional techniques in cardiac surgery patients.^8^ The optimal analgesia regimen after open cardiac surgery is unclear.

Ultrasound‐guided transversus thoracis muscle plane (TTMP) block has been advocated by some researchers for cardiac surgery.^9^, ^10^ Our previous study found that bilateral TTMP blocks can provide good perioperative analgesia for patients undergoing open cardiac surgery and promote their post‐operative recovery.^11^ However, single‐shot TTMP blocks may not be able to produce a durable block. Only one case report found that continuous TTMP blocks effectively relieved perioperative pain for 2 days after median sternotomy.^12^ We hypothesized that continuous TTMP block would be associated with a reduced length of hospital stay and more effective analgesia than placebo after cardiac surgery performed via median sternotomy.

## 2 METHODS

This study was approved by the ethics committee of the First Affiliated Hospital of Nanchang University, and written informed consent was obtained from all subjects participating in the trial. Then, it was registered in the Chinese Clinical Trial Registry (registration number ChiCTR2100047755). Our study adheres to CONSORT guidelines.

This double‐blind, randomized, controlled study was performed on patients between the age groups of 20 and 70 years undergoing open cardiac surgical procedures through median sternotomy with American Society of Anaesthesiologists Physical status II‐III. Criteria for exclusion in our trial were as follows: emergency surgery, an allergy to local anaesthetics, congestive heart failure, hepatic or renal failure, a history of drug abuse or chronic pain, psychiatric problems, secondary surgery and inability to provide informed consent. The patients enrolled in our study were randomly divided into two groups; the TTP group received bilateral TTMP blocks with ropivacaine, and the SAL group received the same block with saline.

### 2.1 Randomization and blinding

All enrolled patients were randomized into the TTP group or SAL group with either ropivacaine or saline using computer‐generated codes, and the group allocation was kept in a sealed envelope by the first investigator. The saline and ropivacaine, which looked identical, were prepared by the second investigator, who was blinded to the grouping. The blinded third investigator was responsible for administering the injectate. Post‐operative data were collected by another researcher. The patients, surgeons, anaesthesiologists, intensive care unit staff, nurses and other investigators were blinded to the medication assignments. This was a double‐blind, randomized, controlled study.

### 2.2 Surgery and anaesthesia

All the patients in our trial underwent continuous intraoperative monitoring of central temperature, invasive arterial blood pressure, electrocardiography, oxyhaemoglobin saturation, end‐tidal carbon dioxide, central venous pressure and urine output. General anaesthesia was induced with 0.1 mg/kg midazolam, 0.8 μg/kg sufentanil, 0.3 mg/kg etomidate and 0.15 mg/kg cisatracurium for tracheal intubation. Anaesthesia was maintained with sufentanil, propofol and cisatracurium according to the clinical needs following induction in both groups, and the BIS was maintained between 45 and 55 in all patients. Patient‐controlled analgesia with intravenous sufentanil was used for post‐operative analgesia, and 50 mg flurbiprofen axetil was injected intravenously at 6 h intervals as a supplementary analgesic according to the demands of the patients. All surgeries were performed by the same group of surgeons in our trial. After the operation, the patients were sent to the cardiac surgery ICU as scheduled.

### 2.3 Ultrasound‐guided continuous TTMP block

**FIGURE 1:**
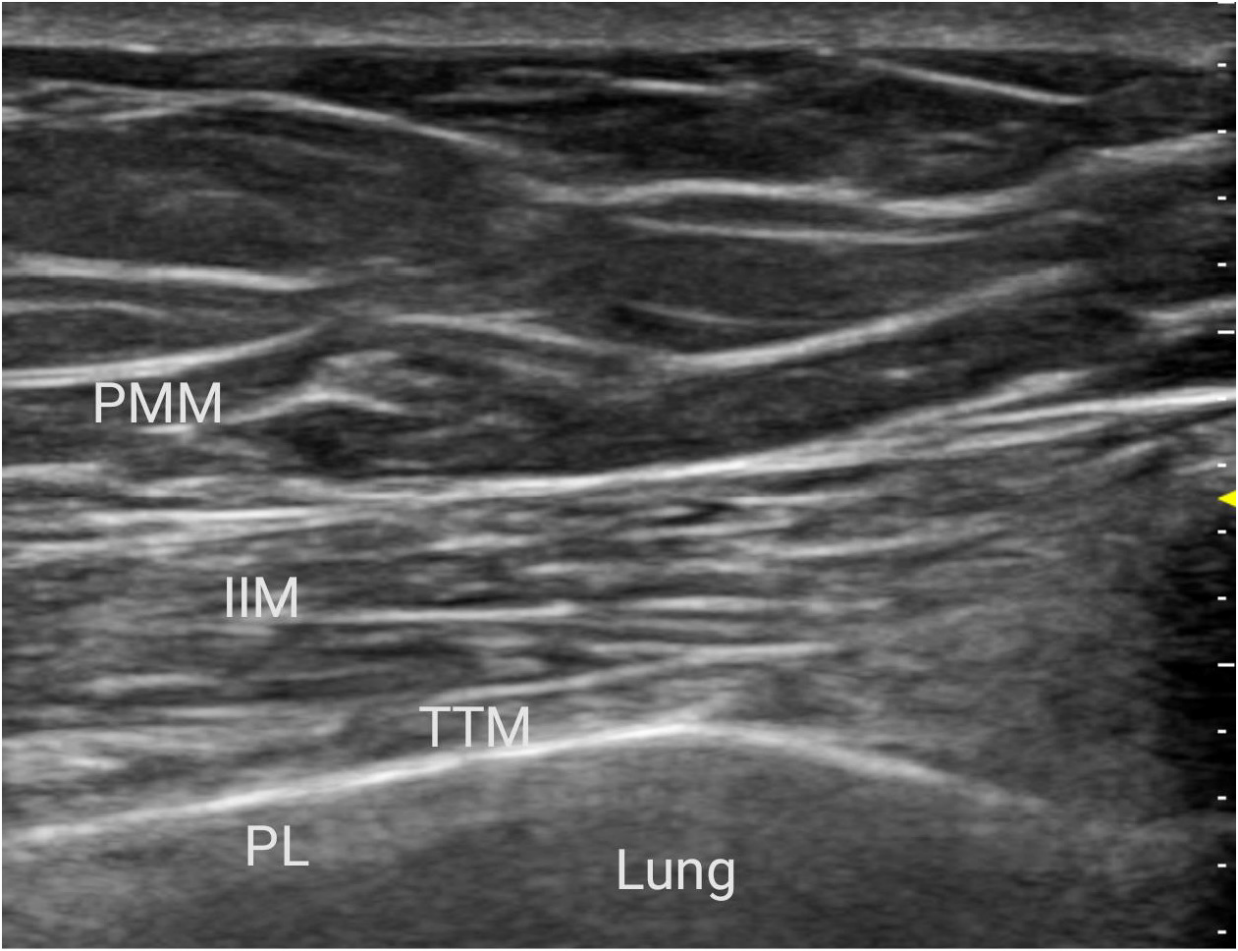
Ultrasound image of transversus thoracis muscle plane block. IIM, internal intercostal muscle; PL, pleura; PMM, pectoralis major muscle; TTM, transversus thoracis muscle.

We performed continuous bilateral TTMP blocks prior to intubation in the operating room using a high‐frequency linear ultrasound probe (Mindray, Shenzhen, China). TTMP blocks were administered bilaterally by injection of 0.33% ropivacaine 40 mL in total (20 mL injected into each side) into the fascial plane between the transversus thoracic muscle and the intercostal muscle between the fourth and fifth connecting at the sternum (Figure 1).^13^ After the injections, a multiorifice catheter was inserted through the needle/cannula bilaterally. For the next 3 days, ropivacaine 0.2% was infused at a rate of 8 mL/h into each catheter, and 6 mL (3 mL injected into each side) ropivacaine 0.2% lock out time was administered for 30 min 3 days post operatively.^10^ All nerve blocks were completed by the same skilled anaesthesiologist within 20 min.

### 2.4 Study endpoints

The primary endpoint was post‐operative pain at 4, 8, 16, 24, 48 and 72 h after extubation at rest and during exercise. The secondary outcome measures included analgesia requirements (sufentanil and flurbiprofen axetil consumption); time to extubation; length of stay in the ICU; incidence of post‐operative nausea and vomiting (PONV); time until return of bowel function; time to mobilization; incidence of reintubation; length of hospital stay; time to urinary catheter removal; incidence of sternal wound infection (evaluated by a surgeon until 60 days after surgery) and possible complications such as ropivacaine allergy and haematomas. The incidence of PONV was calculated based on the need for post‐operative antiemetic agents after surgery. Post‐operative pain was measured using the Numerical Rating Scale (NRS) score, with scores ranging from 0 (no pain) to 10 (worst pain imaginable). Data were recorded by an assistant helping with the experiment who was also blinded to the experimental grouping.

### 2.5 Statistical analysis

The authors calculated the patient sample size of our trial based on a pilot study (*n* = 10 patients per group) that compared the length of hospital stay. A sample size of 46 patients in each group was required with a type I error of *α* = 0.05, a type II error of *β* = 0.1 and a power of 90%. We finally included 20% more patients for analysis to compensate for possible dropout in our trial (*n* = 55 in each group).

Pain intensity after extubation was compared between the TTP group and SAL group with repeated‐measures (two‐way) analysis of variance. Student's *t*‐test was used to assess intergroup differences with a normal distribution, whereas the Wilcoxon Mann–Whitney test was used to assess nonnormally distributed data. The skewness and Kurtosis tests were used to test the normal distribution of continuous data. The chi‐square or Fisher's exact test was used to analyse categorical data. Statistical significance was defined as *p* < 0.05.

## 3 RESULTS

**FIGURE 2:**
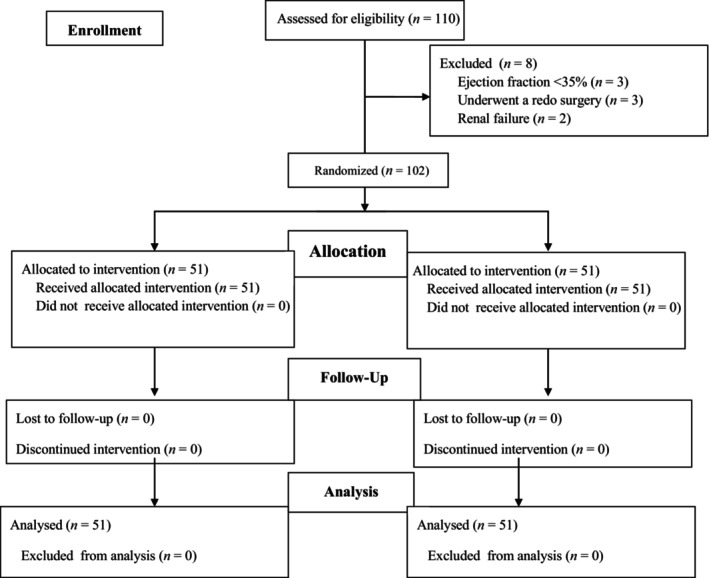
Patient flow diagram.

A total of 110 patients were randomized in our trial. Eight patients were excluded after randomization (four from each group), leaving 102 patients (51 in each group) for data analysis (Figure 2). Baseline characteristics showed no statistically significant differences between the TTP group and the SAL group (Table 1).

**TABLE 1:** Patient demographics and surgical characteristics.

	SAL group (*n* = 51)	TTP group (*n* = 51)	*p*‐Value
Age (year)	53 ± 15	55 ± 14	0.641
Height (cm)	172 ± 10	171 ± 13	0.732
Weight (Kg)	62 ± 17	60 ± 15	0.532
Sex ratio (M/F)	28/23	24/27	0.564
ASA classification (II/III)	22/29	25/26	0.612
Duration of surgery (min)	191 ± 37	187 ± 42	0.542
Size of incision (cm)	21 ± 4	22 ± 3	0.672
Intraoperative urine output (mL)	890 ± 240	850 ± 220	0.542
Intraoperative bleeding volume (mL)	750 ± 240	720 ± 300	0.672
Intraoperative crystalloids (mL)	820 ± 160	790 ± 180	0.752
Intraoperative colloids (mL)	1500 ± 120	1600 ± 120	0.732
Procedure			0.523
Mitral valve replacement	29	25	
Aortic valve replacement	22	26	

**FIGURE 3:**
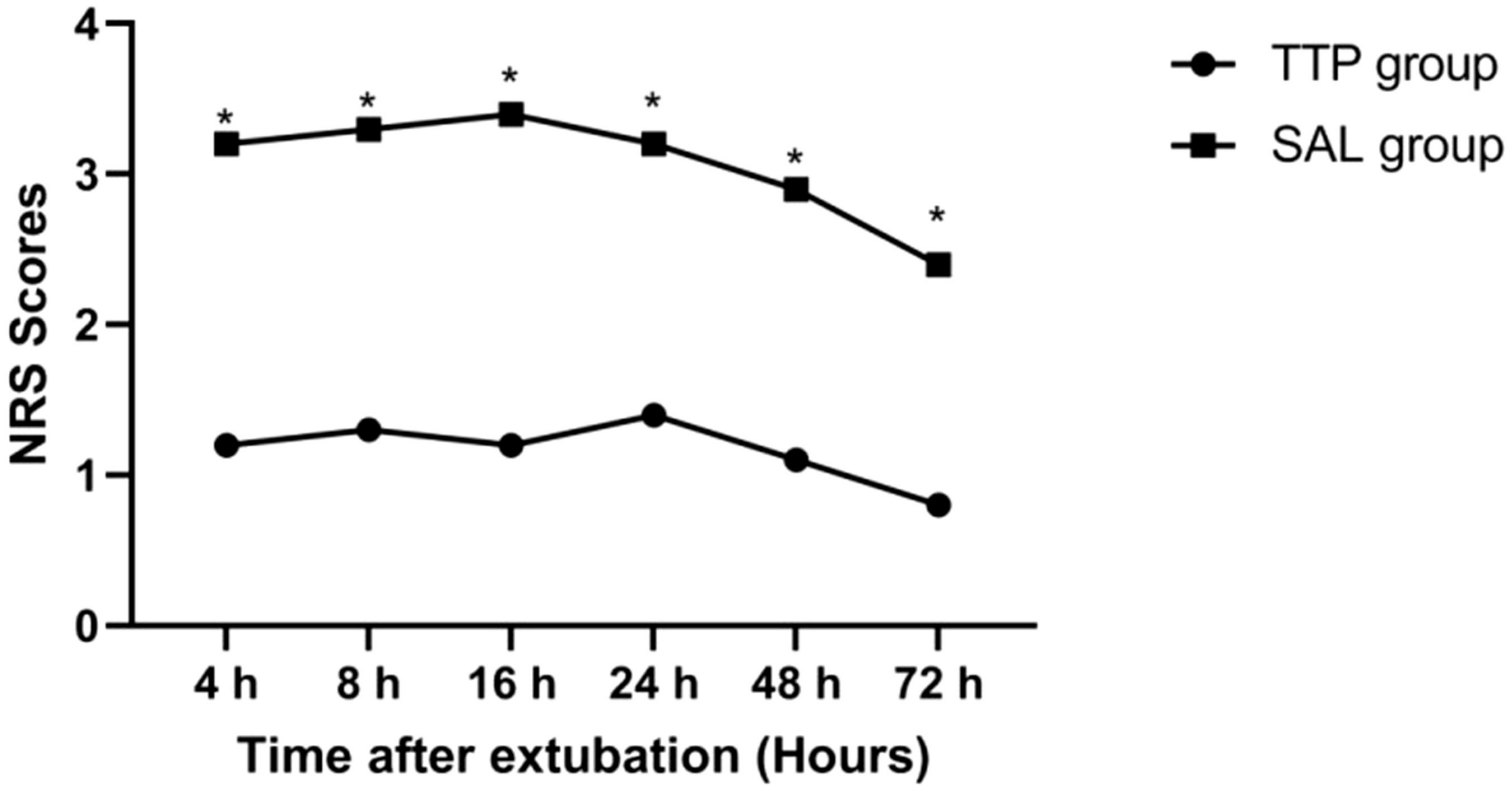
Pain intensity at rest after extubation which was measured using the verbal numerical scale (NRS) score. *p < 0.05 considered statistically significant.

**FIGURE 4:**
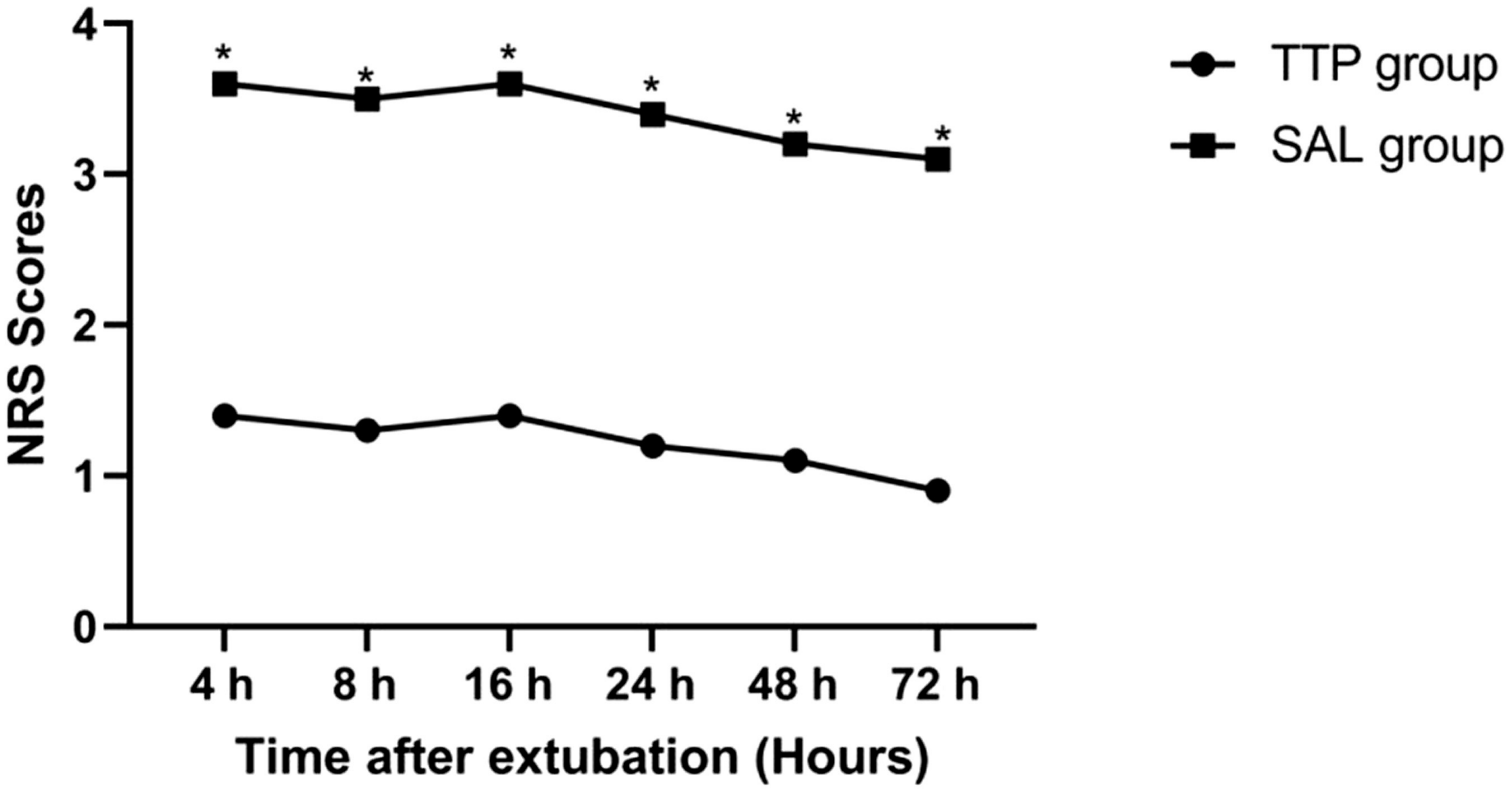
Pain intensity at movement after extubation which was measured using the verbal numerical scale (NRS) score. *p < 0.05 considered statistically significant.

The lengths of stay in the ICU and hospital were significantly longer in the SAL group than in the TTP group (Table 2). NRS scores at rest and during exercise were significantly lower in the TTP group than in the SAL group at all time points (Figures 3 and 4). The TTP group needed less intraoperative and post‐operative sufentanil and post‐operative dynastat than the SAL groups (Table 2). Time to extubation, time to first flatus, time until mobilization and time until urinary catheter removal were significantly shorter in the TTP group than in the SAL group (Table 2). The incidence of PONV was significantly lower in the TTP group (Table 2). There was no statistically significant differences in the incidence of reintubation between the TTP group and the SAL group (Table 2). There were no complications related to TTMP in our study.

**TABLE 2:** Intra‐ and post‐operative clinical outcomes.

	SAL group (*n* = 51)	TTP group (*n* = 51)	*p*‐Value
Intraoperative sufentanil consumption (μg)	115 ± 35	65 ± 12	<0.01
Post‐operative sufentanil consumption (μg)	107 ± 20	55 ± 14	0.02
Flurbiprofen axetil consumption (mg)	300 ± 150	150 ± 100	<0.01
Time to extubation (h)	9.2 ± 2.3	3.4 ± 1.1	0.03
Time to mobilization (h)	32 ± 3	25 ± 4	<0.05
Length of stay in the ICU (h)	26 ± 5	14 ± 3	<0.01
Time until passage of flatus (h)	42 ± 4	31 ± 5	<0.05
Time until urinary catheter removal (h)	45 ± 6	32 ± 3	<0.05
Incidence of PONV (%)	13 (25.4)	4 (7.8)	<0.01
Cardiopulmonary bypass time (min)	60 ± 26	65 ± 20	0.67
Time to drain removal (h)	35 ± 8	38 ± 6	0.84
Incidence of Reexploration for bleeding (%)	0	0	
Incidence of sternal wound infection (%)	0	0	
Incidence of reintubation (%)	1(1.9)	1(1.9)	0.67
Length of hospital stay (h)	205 ± 19	150 ± 28	<0.01

## 4 DISCUSSION

In this study, continuous TTMP block initiated before surgery can reduce the amount of perioperative sufentanil needed and the dosage of post‐operative flurbiprofen axetil and provide effective analgesia for 3 days post operatively in patients who have undergone open cardiac surgery. Furthermore, these results provide guidance for reducing the time to extubation, length of stay in the ICU and hospital stay after surgery.

Ultrasound‐guided TTMP block was first reported by Ueshima.^14^, ^15^, ^16^ TTMP block can cover the anterior branches of intercostal nerves from T2 to T6 and provide effective analgesia in the internal mammary area.^17^ A previous study of 299 patients who underwent TTMP block reported only two cases of ‘mild infection’ around the injection site.^18^ Ueshima et al.^9^ found that, in TTMP block, the spread of ropivacaine between the fourth and fifth ribs is larger than that between the third and fourth ribs. Therefore, the fourth and fifth ribs next to the sternum were used as a reference for the TTMP block for all patients in our study.

Our previous study found that bilateral TTMP blocks can provide good perioperative analgesia for patients undergoing open cardiac surgery and promote their post‐operative recovery.^9^ However, single‐shot TTMP blocks may not be able to produce a durable block. Patients who underwent open cardiac surgery experienced severe and prolonged post‐operative pain, especially at the median sternotomy site, peaking during the first 3 days after the surgery.^19^ Poorly controlled post‐operative pain in open cardiac surgery patients causes haemodynamic instability, hypercoagulability, pulmonary complications, sympathetic activation and increased rates of delirium.^5^ Continuous TTMP blocks effectively relieved perioperative pain at the site of the median sternotomy for 2 days in only one case report. In the current study, we demonstrated that continuous bilateral TTMP blocks provided effective analgesia for open cardiac surgery patients both at rest and during mobilization for 3 days. Moreover, significantly less sufentanil and flurbiprofen axetil were needed in the TTP group, thus reducing the adverse effects of these two drugs. To our knowledge, this was the first randomized controlled study to identify that continuous bilateral TTMP blocks effectively relieves post‐operative pain for 3 days in open cardiac surgery patients.

Neuraxial techniques and paravertebral nerve block can also provide effective post‐operative analgesia for patients undergoing open cardiac surgery.^20^ However, they are not routinely performed in cardiac surgery due to concerns of haemorrhage and haematoma after coagulopathy and heparinization.^18^ Serratus plane block and pectoral nerve block have been used in patients undergoing breast and thoracic surgeries to treat postsurgical pain.^21^ However, these blocks cannot cover the anterior branches of intercostal nerves, which could provide effective analgesia in the internal mammary area. Intercostal nerve block can also provide good perioperative analgesia for open cardiac surgery patients, and it is relatively simple to complete because osseous landmarks are readily palpable and easier to localize with ultrasound guidance.^22^ However, fascial plane blocks are simple and effective alternatives to intercostal nerve block and provide a longer duration of post‐operative analgesia while avoiding multiple injections.^23^

The transversus thoracic muscle is very thin and close to the pleura, which increases the risk of pneumothorax in the TTMP block. The internal mammary artery runs between the intercostal muscle and transversus thoracic muscle, and patients with TTMP block is at risk for internal mammary artery injury or haematoma. Moreover, patients undergoing coronary artery bypass grafting could have tissue disruption in the TTMP due to internal mammary artery harvest, making the transverse thoracic muscle more difficult to recognize.^24^ Therefore, great care should be taken when performing TTMP blocks. However, there were no adverse events related to continuous TTMP blocks in our study, such as haematoma, artery puncture, ropivacaine allergy, pneumothorax, infections, local anaesthesia toxicity and catheter dislodgment or failed block. Therefore, ultrasound‐guided continuous TTMP blocks are novel, effective, promising and safe for regional analgesia in cardiac surgery patients.

High‐dose sufentanil is most commonly used in cardiac surgery patients with haemodynamic stability to provide effective post‐operative analgesia,^25^ but sufentanil can cause adverse effects, including respiratory depression, sedation, ileus, nausea, vomiting, drowsiness and prolonged ICU stays.^26^ In the present study, we found that continuous TTMP blocks decreased the perioperative sufentanil dosage without causing any adverse events. Time to extubation, time to first flatus, time until mobilization and time until urinary catheter removal were significantly shorter in the TTP group than in the SAL group, and the difference was probably caused by the use of a minimal amount of sufentanil and the good analgesic effect of continuous TTMP blocks in open cardiac surgery patients. Therefore, an important part of the enhanced recovery of open cardiac surgery patients in the TTP group may be the minimal amount of sufentanil used.^10^

There are several limitations to this study. First, the volume and concentration of continuous TTMP blocks used in our study were based on previous research.^10^ In further trials, the optimum capacity and concentration of the continuous TTMP blocks in open cardiac surgery patients should be evaluated. Second, effective post‐operative pain relief may prevent the development of chronic pain,^27^ but we did not observe the effect of this block on post‐operative chronic pain. Third, the sample size of our study is small, and larger sample studies are needed to verify its effectiveness in the future.

In conclusion, this study showed that ultrasound‐guided continuous TTMP blocks in open cardiac surgery patients can provide effective post‐operative pain relief for 3 days, thus reducing the dose of sufentanil and flurbiprofen axetil needed, decreasing the mechanical ventilation time and the time to first flatus and reducing the lengths of stay in the ICU and hospital.

## AUTHOR CONTRIBUTIONS

**Yanping Zhan:** Conceptualization (equal); data curation (equal); formal analysis (equal); investigation (equal); methodology (equal); resources (equal); software (equal); supervision (equal); validation (equal). **Lei Li:** Conceptualization (equal); data curation (equal); formal analysis (equal); funding acquisition (equal); investigation (equal); resources (equal); software (equal); validation (equal); visualization (equal). **Shibiao Chen:** Investigation (equal); methodology (equal); resources (equal); software (equal); supervision (equal). **Yongbao Peng:** Conceptualization (equal); data curation (equal). **Yang Zhang:** Conceptualization (equal); data curation (equal); formal analysis (equal); funding acquisition (equal); investigation (equal); methodology (equal); resources (equal); supervision (equal); validation (equal); writing – review and editing (equal).

## FUNDING INFORMATION

The project was funded by the Department of Science and Technology of Jiangxi Province 20212BAG70034, 20181BBG78055 and the National Natural Science Foundation of China (82060023 and 82360385). The Rapid Service Fee was funded by the authors.

## CONFLICT OF INTEREST STATEMENT

None of the authors have any conflicts of interest to declare.

## Data Availability

Data available upon request from the authors.The data that support the findings of this study are available from the corresponding author upon reasonable request.
