# Correction to “Randomized Clinical Trial of Continuous Transversus Thoracis Muscle Plane Block for Patients Undergoing Open Heart Valve Replacement Surgery”

**DOI:** 10.1111/jcmm.70648

**Published:** 2025-06-05

Zhan Y, Li L, Chen S, Peng Y, Zhang Y. Randomized Clinical Trial of Continuous Transversus Thoracis Muscle Plane Block for Patients Undergoing Open Heart Valve Replacement Surgery. *J Cell Mol Med*. 2024; 28:e18184. doi: 10.1111/jcmm.18184.

In the article, references 12, 13, 14, 17, and 18 were incorrect and should be replaced.

The incorrect references are:

12. Ueshima H, Otake H. Continuous transversus thoracic muscle plane block is effective for the median sternotomy. *J Clin Anesth*. 2017;37:174.

13. Ueshima H, Otake H. Where is an appropriate injection point for an ultrasound‐guided transversus thoracic muscle plane block? *J Clin Anesth*. 2016;33:190‐191.

14. Ueshima H, Takeda Y, Ishikawa S, Otake H. Ultrasound‐guided transversus thoracic muscle plane block: a cadaveric study of the spread of injectate. *J Clin Anesth*. 2015;27:696.

17. Ueshima H, Hara E, Marui T, Otake H. The ultrasound‐guided transversus thoracic muscle plane block is effective for the median sternotomy. *J Clin Anesth*. 2016;29:8.

18. Ueshima H, Otake H. Ultrasound‐guided transversus thoracic muscle plane block: complication in 299 consecutive cases. *J Clin Anesth*. 2017;41:60.

The correct references are:

12. Avneet Singh, Indumati, Dheeraj Kapoor, Suman Dhillon, Jasmine K Narula, Sidharth Garg. Continuous Bilateral Transversus Thoracic Muscle Plane Block: An Analgesia Boon for Scoliotic Patients Undergoing Cardiac Surgery. *Ann Card Anaesth*. 2024 Jan 1;27(1):61‐64.

13. Samerchua Artid, Leurcharusmee Prangmalee, Supphapipat Kittitorn, Unchiti Kantarakorn, Lapisatepun Panuwat, Maikong Naraporn, Kantakam Perada, Navic Pagorn, Mahakkanukrauh Pasuk. Optimal techniques of ultrasound‐guided superficial and deep parasternal intercostal plane blocks: a cadaveric study. *Region Anesth Pain* M. 2024‐05‐07;49(5):320‐325.

14. Harbell Monica W, Langley Natalie R, Seamans David P, Kraus Molly B, Carey Frederick J, Craner Ryan C.Deep parasternal intercostal plane nerve block: an anatomical study. *Region Anesth Pain* M. 2024‐03‐04;49(3):179‐183.

17. Mohamed Ahmed Hamed, Maged Labib Boules, Mina Mahrou Sobhy, Mahdy Ahmed Abdelhady.The Analgesic Efficacy of Ultrasound‐Guided Bilateral Transversus Thoracic Muscle Plane Block After Open‐Heart Surgeries: A Randomized Controlled Study.*J Pain Res*. 2022‐01‐01;15:675‐682.

18. Jian‐Jun Xue, Yi‐Yang Cui, Jason W Busse, Long Ge, Ting Zhou, Wei‐Hua Huang, Sheng‐Shuang Ding, Jie Zhang, Ke‐Hu Yang. Transversus thoracic muscle plane block for pain during cardiac surgery: a systematic review and meta‐analysis. *Int J Surg*. 2023 Aug 1;109(8):2500‐2508.

The online version of the article was corrected.

We apologize for these errors.

